# A Robust Panel Based on Mitochondrial Localized Proteins for Prognostic Prediction of Lung Adenocarcinoma

**DOI:** 10.1155/2021/7569168

**Published:** 2021-09-09

**Authors:** Weifeng Chen, Jingyao Wang, Qiumei Zhao, Dandan Liu, Donglin Sun, Ningxia Xie, Haohao Zhang, Deji Ye, Chaoyang Li, Yongzhong Liu, Xiaoren Zhang

**Affiliations:** ^1^Affiliated Cancer Hospital and Institute of Guangzhou Medical University; Key Laboratory for Cell Homeostasis and Cancer Research of Guangdong Higher Education Institutes, State Key Laboratory of Respiratory Disease, Guangzhou Medical University, Guangzhou 510000, China; ^2^State Key Laboratory of Oncogenes and Related Genes, Shanghai Cancer Institute, Renji Hospital, Shanghai Jiao Tong University School of Medicine, Shanghai 200032, China

## Abstract

Due to high energy and material metabolism requirements, mitochondria are frequently active in tumor cells. Our study found that the high energy metabolism status is positively correlated with the poor prognosis of patients with lung adenocarcinoma. We constructed a scoring system (mitoRiskscore) based on the gene expression of specific mitochondrial localized proteins through univariate and LASSO cox regression. It has been shown that high mitoRiskscore was correlated with a shorter survival time after surgery in patients with lung adenocarcinoma. Compared with the typical TNM grading system, the mitoRiskscore gene panel had higher prediction accuracy. A vast number of external verification results ensured its universality. Additionally, the mitoRiskscore could evaluate the metabolic pattern and chemotherapy sensitivity of the tumor samples. Lung adenocarcinoma with higher mitoRiskscore was more active in glycolysis, and oxidative phosphorylation expression of proliferation-related pathway genes was also significantly upregulated. In contrast, patients with low mitoRiskscore had similar metabolic patterns to normal tissues. In order to improve the accuracy of prediction ability and promote clinical usage, we developed a nomogram that combined mitoRiskscore and clinical prognostic factors to predict the 3-year, 5-year, and 10-year survival rates of patients. We also performed in vitro experiments to verify the function of the key genes in the mitoRiskscore panel. In conclusion, the mitoRiskscore scoring system may assist clinicians to judge the postoperative survival rate and chemotherapy of patients with lung adenocarcinoma.

## 1. Introduction

In recent decades, lung cancer is a common occurrence and the leading cause of cancer-related deaths. According to epidemiological data in 2018, there were 2.09 million lung cancer cases and 1.76 million deaths worldwide [1]. Lung adenocarcinoma (LUAD) accounts for approximately 85% of lung cancers and is the most common histological type. At present, the known factors related to the occurrence of LUAD include gender, age, smoking, environment, and heredity [2]. With the rise of medical technology, lung cancer treatment is gradually increasing, from simple surgical resection to surgery combined with adjuvant chemotherapy, immunotherapy, targeted drug therapy, and biological therapy [3]. Although we have made innovations in lung cancer treatment, LUAD's annual mortality rate has not decreased as expected. It may be due to our lack of assessment of LUAD patients' survival status after surgery [4]. If we can accurately predict patients' survival rate after surgery and each patient's treatment preference, clinicians can provide patients with accurate postoperative treatment. For example, patients with low predicted survival rates should be reviewed more frequently and treated with high-dose drugs; for patients with high predicted survival rates, more conservative treatments should be used to improve patients' quality of life.

For the prognostic evaluation of LUAD, although there are some methods (such as age, stage, and grade) to predict patients' survival rate, these prediction methods are not accurate enough. It is generally believed that patients with early lung cancer have a good prognosis, but the five-year survival rate after surgical intervention fluctuates by 34% (10% to 44%) [5]. Besides, age and stage cannot explain the evaluation basis of patients' resistance to immunotherapy [6]. Therefore, we need to develop more methods to accurately predict the postoperative survival and treatment preferences of LUAD patients.

Mitochondria are the most important places for energy and matter conversion in organisms. When a cell undergoes a malignant transformation, the mitochondrial metabolism mode, transport mode, kinetics, and response to oxidative stress will change significantly [6]. Among them, the intensity of glucose metabolism in cancer cells is significantly increased to produce more intermediate metabolites [7]. Due to the increased energy demand of cancer cells, the mitochondrial oxidative phosphorylation of (OXPHOS) is also significantly improved [8]. In addition, the decomposition of fatty acids and glutamine in cancer cells was also enhanced to provide intermediates for mitochondrial productivity [9]. The high metabolism of mitochondria in cancer cells produces excessive amounts of reactive oxygen species (ROS), cause the death of normal cells, thereby promoting tumor progression [10]. Abnormal mitochondrial function plays an essential role in the occurrence and development of cancer. From another perspective, we can judge the malignant degree and progress trend of the tumor by evaluating the activity of the mitochondria of the tumor.

We analyzed four LUAD mRNA microarrays and reached the same conclusion. Compared with normal tissues, LUAD has a significant increase in mitochondria's metabolic activity, especially the expression of genes encoding mitochondrial localization protein (mito-protein genes). Besides, we also found that a vast number of mito-protein genes are significantly related to patients' prognosis. Therefore, we established a mitochondrial risk score (mitoRiskscore) to predict the prognosis and treatment preferences of LUAD patients. The robustness of the risk score has been verified in multiple external data sets. It brings the possibility of precise treatment of LUAD patients after surgery.

## 2. Materials and Methods

### 2.1. Data Source and Study Population

The LUAD RNA-seq dataset used in this study came from the Cancer Genome Atlas project (TCGA) [11]. A total of nine microarray datasets of LUAD were downloaded from Gene Expression Omnibus (GEO). Four datasets containing LUAD samples and control samples were used to analyze the abnormal activity of mitochondria in lung cancer (GSE7670, GSE18842, GSE19188, and GSE31210). Six datasets containing prognostic information of patients were used to verify the prediction ability of mitoRiskscore (GSE3141, GSE8894, GSE31210, GSE50081, GSE68465, and GSE72094). The details about the datasets used in this study are presented in Table 1. The self-composed running scripts, together with the processed results of current study, were merged into a repository that is available at https://github.com/XR-Zhang-group/mitoRiskscore.

### 2.2. Pathway Enrichment Analysis

First, the differentially expressed genes between the LUAD samples and the control samples were obtained by using the R package “limma.” Then, the R package “clusterProfiler” [12] was used to perform gene set enrichment analysis (GSEA). The five pathways most related to mitochondrial activity were selected for evaluation. The pathway gene sets (MITOCHONDRIAL PART, HALLMARK FATTY ACID METABOLISM, HALLMARK GLYCOLYSIS, HALLMARK HYPOXIA, HALLMARK OXIDATIVE PHOSPHORYLATION, and HALLMARK REACTIVE OXYGEN SPECIES PATHWAY) were downloaded from the molecular signature database (MSigDB) [13]. Mito-protein genes were obtained from the gene set “MITOCHONDRIAL PART” in the MSigDB. The systematic name of this pathway is M18830.

### 2.3. Preliminary Screening of Prognostic-Related Genes

Univariate Cox proportional hazard regression analysis was performed on each mito-protein gene to screen for genes that were significantly related to the overall survival rate of TCGA-LUAD [14]. Then, the bootstrapping method was used to test the robustness of these genes [15]. 70% of the samples were randomly selected from the cohort, and the survival impact of these genes was evaluated. This process was repeated 1000 times. A gene that had a valid *P* value at all times was considered to have passed the bootstrapping robustness test.

### 2.4. Mito-Protein Gene Panel Generation

Because of the large number of genes that passed the bootstrapping robustness test, it is not convenient for follow-up clinical application. The least absolute shrinkage and selection operator (LASSO) Cox regression analysis was performed on the genes that passed the test. While reducing the number, the genes most related to prognosis were also screened out. Through LASSO analysis, the gene represented by the minimum penalty parameter *λ* will be selected to establish a prognostic risk score formula (mitoRiskscore). We used the “pheatmap” package to visualize the expression of each gene in mitoRiskscore. The Kaplan-Meier method was used to calculate the survival rate, and the log-rank test was performed to evaluate its statistical significance. Univariate and multivariate cox regression analyses were used to test whether the prediction model based on mitoRiskscore was an independent prognostic factor. The time-dependent receiver operating characteristic curve (TDROC) from the “survivalROC” package [16] was applied to test the predictive ability of mitoRiskscore at 1, 3, and 5 years.

### 2.5. Gene Set Variation Analysis (GSVA)

Gene set variation analysis is one of the most commonly used methods for analyzing biological processes [17]. The gene set file “http://c2.cp.kegg.v7.3.symbols.gm” downloaded from MSigDB was used for GSVA, and the “GSVA” package in the R software was used. The significance threshold was set at FDR < 0.05.

### 2.6. Prediction of Chemotherapy Response

The R package “pRRophetic” [18] and the TCGA-LUAD data were used to predict the drug sensitivity of each group of patients. Among them, ridge regression was used to estimate the maximum half-inhibitory concentration (IC50) of the samples, and ten-fold cross-validation was used to evaluate the accuracy.

### 2.7. Cell Culture and Treatment

Human lung adenocarcinoma cells, A549, were cultured in RPMI-1640 medium supplemented with 10% fetal bovine serum, 50 U/mL penicillin, and 50 mg/mL streptomycin. Cells were incubated at 37°C under 5% CO_2_. To knockdown VDAC1, A549 cells were transfected with VDAC1 siRNAs (Tsingke Biotechnology, China) using Lipofectamine 2000 regent (Thermo Fisher Scientific, USA) according to the manufacturer's recommendation. The sequences of the two siRNA were as follows:

siRNA #1-F (5′-3′): GGAUACACUCAGACUCUAATT

siRNA #1-R (5′-3′): UUAGAGUCUGAGUGUAUCCTT

siRNA #2-F (5′-3′): GGAUGGCAAGAACGUCAAUTT

siRNA #2-R (5′-3′): AUUGACGUUCUUGCCAUCCTT

### 2.8. RNA Extraction and qRT-PCR

Total RNA was extracted using TRIzol (Invitrogen, USA). qRT-PCR was carried out with PrimeScript RT reagent Kit and TB Green Premix Ex Taq II kit (TaKaRa, China) according to the manufacturer's protocol. The primers used in the study were as follows:

VDAC1-F1: GCAAAATCCCGAGTGACCCAGA

VDAC1-R1: TCCAGGCAAGATTGACAGCGGT

SLC25A42-F: GCAGCTACTATGGCTTCCGT

SLC25A42-R: TTTCCTTCGGGGTTACGGC

ABAT-F: CAAGGAAAGAGGGCAGAGGG

ABAT-R: GGGTATTTCAGCCGTGGGAA

IVD-F: GTGAGTACATCGGAGCCCTG

IVD-R: TGCTAAAGCCAGGCATACCC

AMT-F: CGAGGCTGGAGGCATCTTAG

AMT-R: ACAGCACTGGTCATGAAGGG

### 2.9. Extracellular Acidification Rate (ECAR) Detection

Seahorse XF glycolysis pressure test kit was used to test the glycolysis ability of cells. On the night before the experiment, A549 cells were transfected into si-VDAC1 or control si-RNA for 48 hours, and the number of cells per well was paved into XF24 test plate for overnight culture. At the same time, the test board will be soaked in the test solution overnight. On the next day, the cells were removed and washed with glycolysis pressure test solution and then cultured in a CO_2_-free incubator for one hour. During the period of cell culture, we prepared the concentration of glucose, oligomycin, and 2-DG. Take out the hydrated test board and add each medicine to the hole as required. Set the experimental template on the XF controller, and the default Mix-Wait-Measure time is 3 min-2 min-3 min. Usually, the basic value is measured three times before adding the drug and then three times after each addition. Put it into the test board and wait for the machine to be calibrated, then replace the utility with the cell culture plate for the ECAR test. Each experiment was repeated three times.

### 2.10. Western Blotting

72 hours after transfection of si-VDAC1, A549 cells were collected and incubated in 200 *μ* L 1∗SDS-PAGE Sample Loading Buffer (Cat No. P0015L), 98°C for 10 minutes until the cells were completely lysis. Proteins were resolved on 10% gel using PAGE gel quick preparation kit (10%, CAT: PG112) and transferred to Trans-blot Turbo nitrocellulose membranes (Bio-Rad). Then, incubate the membrane in 10% milk and seal it for 2 hours. For primary antibody–protein hybridization, membranes were probed with the following antibodies at 4°C overnight (Beyotime): LDHA Rabbit Polyclonal Antibody, CAT: AF0216; COX IV Rabbit Polyclonal Antibody, CAT: AF6549; Hexokinase II Rabbit Polyclonal Antibody, CAT: AF7080; SDHB Rabbit Polyclonal Antibody, CAT: AF7956; and PGK1 Rabbit Monoclonal Antibody, CAT: AF1825. Thereafter, secondary anti-mouse (Beyotime CAT: LF101) or anti-rabbit IgG antibodies (Beyotime CAT: LF102) were incubated for 1 h at room temperature. Protein bands were developed with chemiluminescent reagents (Beyotime) and imaged with a Tanon 5200.

### 2.11. Cell Proliferation Experiment (CCK8)

After 36 hours transfection, the cells were counted and inoculated in a 96-well plate. After 24, 48, and 72 hours of culture, 10 *μ*L CCK8 solution (Beyotime, China) was added to each well. The OD value of each hole was measured.

### 2.12. Wound Healing Assay

After si-VDAC1 or NC mimic transfection, A549 cells grew for 24 hours. When the cell confluence rate was about 70-80%, the cells were scratch-wounded in a straight line. The cells were photographed after wounding and after 36 hours of incubation. The wound healing rate was calculated as the area of cells that migrate from the wound edge into the wound zone.

### 2.13. Transwell Invasion Assays

The cell invasion assay was performed in 24-well plates using filters with an 8.0 *μ*m pore size (Corning, USA) coated with 1: 8 dilution Matrigel (BD Bioscience, USA). Six hundred *μ*L medium containing 10% FBS was added to the lower chamber, and A549 cells in serum-free medium were seeded into the upper chambers. After 36 hours of incubation, the cells were fixed with 4% paraformaldehyde and stained with 0.5% crystal violet. Cells that had invaded the lower surface were captured and evaluated in 6 randomly selected areas.

### 2.14. Statistical Analyses

Unpaired Student's *t*-test or one-way ANOVA test was used to compare normally distributed data. Nonnormally distributed data were performed using the Mann–Whitney *U* test or Kruskal-Wallis test. The prognostic nomogram's construction used R package “rms” and followed by Iasonos' guide [19]. All statistical tests and visual analyses were performed using the R software (version 4.0.3) or GraphPad Prism 6.0 (GraphPad Software, USA).

## 3. Results

### 3.1. GSEA Analysis of Mitochondrial Activity in LUAD

**Figure 1:**
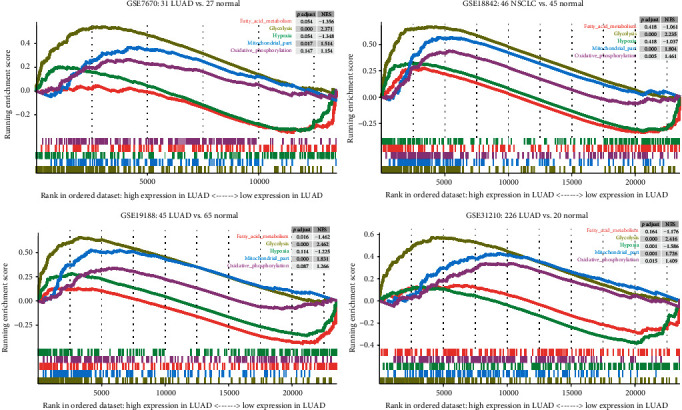
GSEA analyzes the difference in mitochondrial activity between lung adenocarcinoma and normal controls. Five gene sets related to mitochondrial activity from four GEO cohorts were analyzed. The curve above the enrichment score of 0 points indicates that the gene set is activated in lung adenocarcinoma. A curve below 0 points indicates that it is more active in the control group than in lung adenocarcinoma. *p*.adjust: adjusted *p* value; NES: normalized enrichment score.

In order to explore the mitochondrial activity in lung cancer, we downloaded several mitochondrial-related pathways from MSigDB, including the complete mito-protein genes, fatty acid metabolism, glycolytic pathways, hypoxia and high expression genes, and oxidative phosphorylation. Next, the changes of these pathways in the four microarrays were analyzed, namely, GSE7670: 31 LUAD vs. 27 normal, GSE18842: 46 LUAD vs. 45 normal, GSE19188: 45 LUAD vs. 65 normal, and GSE31210: 226 LUAD vs. 20 normal. In all four data sets (Figure 1), glycolytic activity in LUAD was the most significantly increased. Mito-protein genes and oxidative phosphorylation are also upregulated. However, fatty acid metabolism and hypoxia are indeed downregulated in LUAD. It shows that lung cancer cells are not hypoxic, and fatty acid metabolism is not vigorous. The results of GSEA indicate that there is a significant correlation between LUAD and mitochondrial activity and provide a theoretical basis for the development of mito-protein genes to predict the prognosis of LUAD patients.

### 3.2. Relationship between Mito-Protein Genes and Prognosis

We used TCGA-LUAD mRNA-seq data and clinical data to perform univariate Cox regression analysis to examine the relationship between mito-protein genes and patients' prognosis. As shown in Supplementary Figure S1, we found 199 genes that are significantly related to the prognosis of LUAD patients (*p* < 0.05, HR < −1, or HR > 1) and passed the bootstrap test. Among them, 90 genes have hazard ratios less than 1, indicating that patients who overexpress these genes have a longer survival time. In comparison, 109 genes with hazard ratios greater than 1 have the opposite meaning.

### 3.3. Construction of a Prognostic Risk Panel

**Figure 2:**
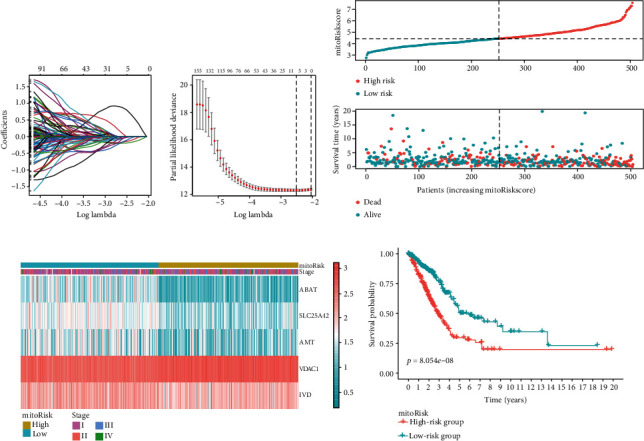
Construction of the mitoRiskscore prediction panel. (a) The convergence of the LASSO cox regression coefficients. (b) A coefficient profile plot of the log (lambda) in the LASSO model. (c) The distribution of mitoRiskscore and the survival status of patients with different scores. (d) Heat map of the expression profiles of the mitoRiskscore gene panel. (e) Kaplan-Meier curves of overall survival for patients in high-risk group and low-risk group.

The 199 robust prognostic genes were subjected to LASSO Cox regression analysis for dimension reduction. The convergence of the regression coefficients is shown in Figure 2(a). Through a random sampling method of 10 cross-validation, we found that the model constructed from 5 genes performed best (Figure 2(b)). Based on the model correlation coefficient, we constructed a mito-protein gene panel to predict the prognosis of LUAD patients.

(1) $$\begin{array}{c} \text{mitoRiskscore}=0.8056*{\text{Exp}}_{\text{VDAC}1}-0.1433*{\text{Exp}}_{\text{SLC}25\text{A}42}-0.1913*{\text{Exp}}_{\text{ABAT}}-0.1083*{\text{Exp}}_{\text{IVD}}-0.0255*{\text{Exp}}_{\text{AMT}} \end{array}$$

We calculated the mitoRiskscore score for each LUAD patient. Patients were divided into two groups (high-risk group and low-risk group) according to mitoRiskscore by using the median of the cohort as the cut-off value. The distribution of mitoRiskscore and patient survival status is shown in Figure 2(c).  Figure 2(d) shows the expression of these 5 genes in the two groups of patients. The Kaplan–Meier curve indicated that the survival rate of patients in the high-risk group was significantly lower (Figure 2(e)).

### 3.4. Independent Prognostic Value of the MitoRiskscore

**Figure 3:**
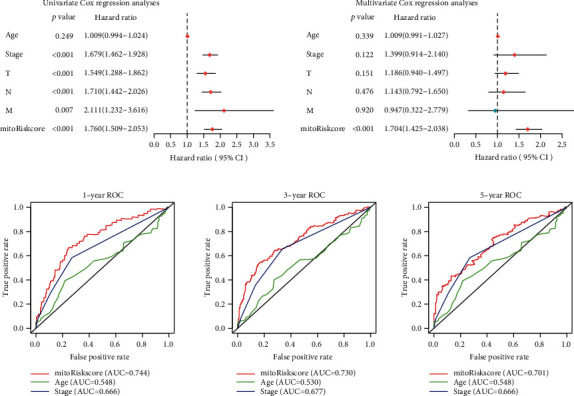
Verification of the independent prognostic value of the mitoRiskscore. (a) Forest plots of the univariate and multivariate Cox regression analyses among mitoRiskscore and clinical factors. (b) Time-dependent receiver operating characteristic curves at 1-year, 3-year, and 5year. AUC: Area Under Curve.

Univariate and multivariate Cox regression analyses demonstrated that mitoRiskscore was a strong independent risk factor for the overall survival of LUAD patients (Figure 3(a)). The predictive power of mitoRiskscore (*p* < 0.001, HR = 1.704, 95% CI = 1.425-2.038) was even higher than age and TMN stage. In the ROC analysis of one year, three years, and five years (Figure 3(b)), mitoRiskscore showed higher predictive ability than the TNM stage and age. The accuracy of mitoRiskscore's judgment of the patient's survival status reached more than 0.7 in all three ROC analyses. These data indicated that mitoRiskscore is an independent prognostic factor and may predict patient prognosis more accurately than existing clinical parameters.

### 3.5. External Verification of the MitoRiskscore

**Figure 4:**
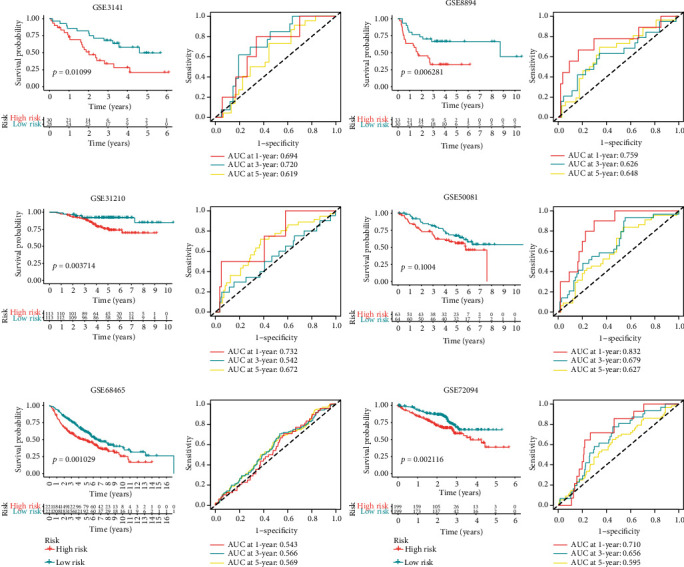
Results of external verification of mitoRiskscore using six microarray cohorts. Each cohort was equally divided into high- and low-risk group based on the value of mitoRiskscore. Kaplan-Meier analysis and time-dependent receiver operating characteristic curves of each cohort are displayed.

A robust prediction panel is universally applicable to other cohorts. In order to verify whether mitoRiskscore applies to other LUAD cohorts, we used external data for verification. We enrolled six cohorts with more than 50 samples each to ensure the reliability of the analysis results (GSE3141, GSE8894, GSE31210, GSE68465, GSE72094, GSE50081). We performed the same procedure to divide patients into high-risk and low-risk groups based on mitoRiskscore. The detailed data of the GEO cohorts performed in the external verification was uploaded in https://github.com/XR-Zhang-group/mitoRiskscore. Surprisingly, in all validation cohorts, patients in the high-risk group showed a higher mortality rate. Except for GSE50081 (*p* = 0.1004), the other results were statistically significant (Figure 4). Besides, we also use mitoRiskscore to predict patients' 1-year, 3-year, and 5-year survival rates. In most ROC analyses, mitoRiskscore's AUC reached 0.6 or more. The above data showed that mitoRiskscore has specific practical application value in predicting the prognosis of LUAD patients.

### 3.6. Identify the Metabolic Patterns of Patients with Different MitoRiskscore

**Figure 5:**
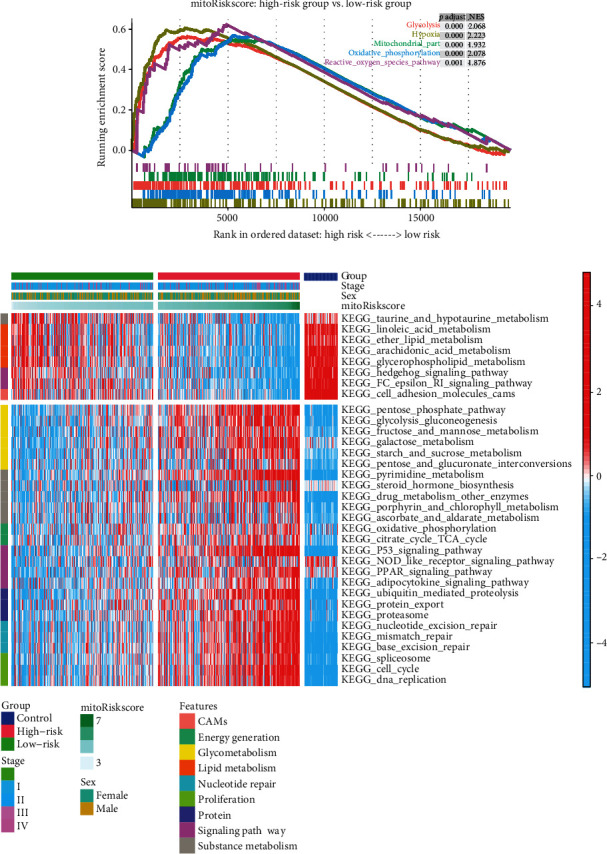
The mitochondrial activity and metabolic patterns of patients with different mitoRiskscore. (a) Enrichment plot of the five gene sets related to mitochondrial activity between the high- and low-risk groups in TCGA-LUAD using GSEA analysis. (b) Heat map showing the activation status of the biological processes in patients with different mitoRiskscore using GSVA analysis.

In order to determine the metabolism and mitochondrial activity in patients with high mitoRiskscore and patients with low scores, we performed a GSEA analysis (Figure 5(a)). The results showed that fatty acid metabolism, glycolysis, hypoxia, oxidative phosphorylation, ROS pathway, and mito-protein genes were significantly activated in patients with high mitoRiskscore compared with patients with low scores. We performed GSVA enrichment analysis to determine the biological behavior characteristics of patients with different mitoRiskscore (Figure 5(b)). The results showed that LUAD with high mitoRiskscore scores has higher glycometabolism and energy generation, as well as higher proliferation activity and nucleotide repairment. In addition, we found that the energy metabolism pattern of low mitoRiskscore LUAD was similar to that of the adjacent cancer controls. They are not active in glycometabolism and oxidative phosphorylation, but they had more lipid metabolism than LUAD with high mitoRiskscore. It confirmed that mitoRiskscore could distinguish LUAD patients with different metabolic patterns.

### 3.7. MitoRiskscore Predicts Therapeutic Benefit of Chemotherapy

**Figure 6:**
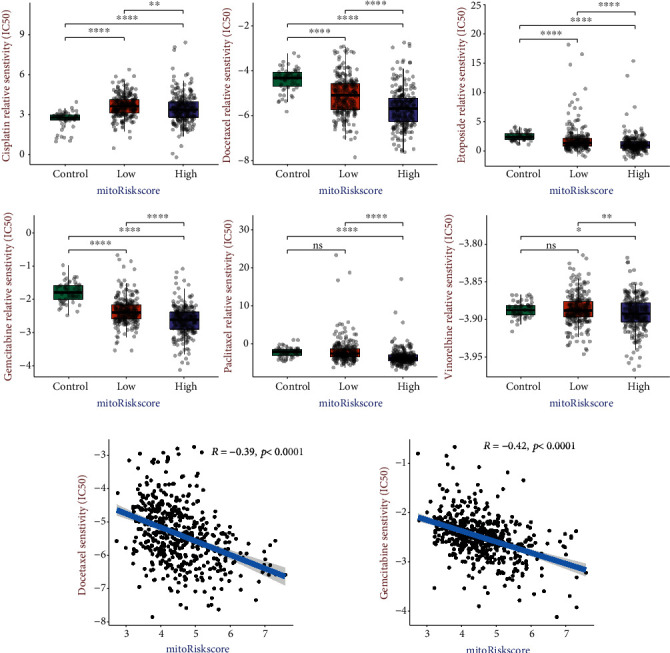
Differences in sensitivity of patients with different mitoRiskscore to chemotherapy. (a) The box plots of the estimated IC50 for commonly used chemotherapy drugs. (b) Correlation analysis between IC50 of two drugs and mitoRiskscore. ^∗^*p* < 0.05, ^∗∗^*p* < 0.01, ^∗∗∗^*p* < 0.001, ^∗∗∗∗^*p* < 0.0001.

Adjuvant chemotherapy is the main treatment strategy for LUAD patients after surgery. The effectiveness of chemotherapy drugs is often related to the metabolism of tumor cells. We selected six commonly used chemotherapy drugs for LUAD patients and evaluated the chemotherapy sensitivity of patients with different mitoRiskscore. All LUAD samples were divided into high-risk and low-risk groups based on the mitoRiskscore. 59 paracancerous tissues in the TCGA-LUAD dataset were used as a control group. The differences in chemotherapy sensitivity between the three groups were then compared (Figure 6(a)). For cisplatin, the IC50s of the LUAD groups were higher than that of the control group. For vinorelbine and paclitaxel, there were no significant difference in IC50s between the LUAD groups and the control group. The IC50s of etoposide in the LUAD groups were lower than that in the control group. But at the same time, many scattered points were displayed higher than the control group's average. It means that etoposide's effectiveness varies greatly, and some patients are not sensitive to it. Furthermore, the LUAD groups were more sensitive to docetaxel and gemcitabine than the control group. Then, we did correlation analysis on the relationship between the IC50 of these two drugs and mitoRiskscore (Figure 6(b)). The results showed that the higher the mitoRiskscore, the lower the IC50 (*R* = −0.39 and *R* = −0.42). It showed that docetaxel and gemcitabine may be two effective drugs for high mitoRiskscore patients.

### 3.8. Develop a Prognostic Nomogram Based on MitoRiskscore

**Figure 7:**
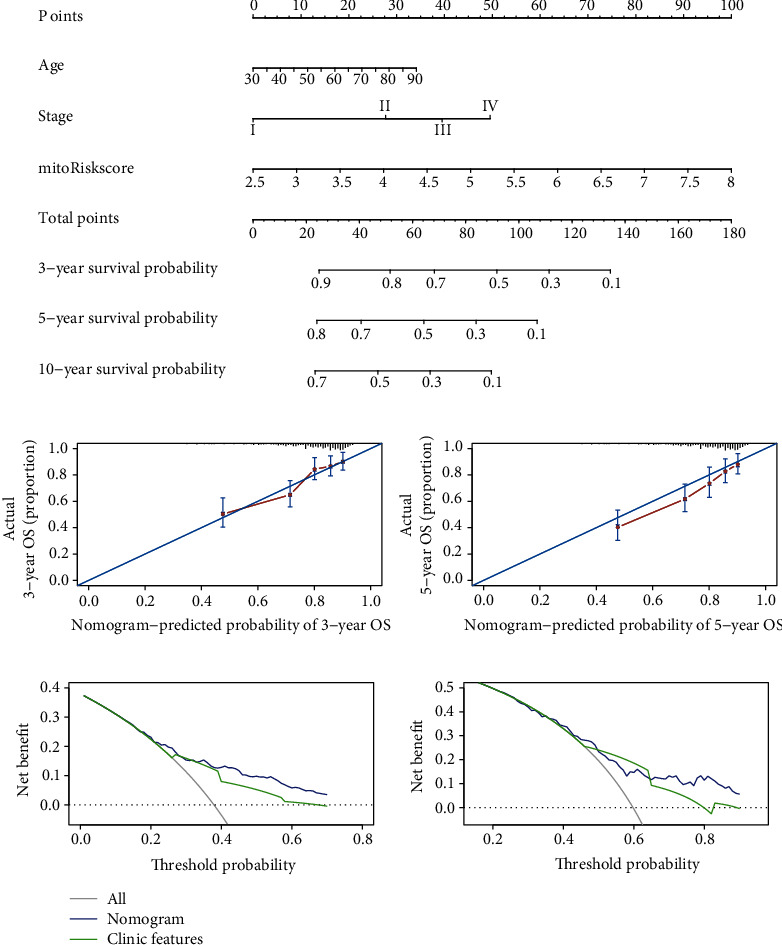
The establishment and verification of prognostic nomogram based on mitoRiskscore. (a) A nomogram for predicting 3-, 5-, and 10-year survival possibilities of individual LUAD patients. (b) Plots depict the calibration of the nomogram based on mitoRiskscore in terms of consistency between predicted and observed 3- and 5-year outcomes. (c) Decision curve analyses of the nomogram for 3- and 5-year risk.

In order to improve the accuracy of prognosis and facilitate clinical usage, we developed a nomogram that integrates mitoRiskscore and clinical prognostic factors to predict the 3, 5, and 10-year survival rate of patients (Figure 7(a)). The sum of each factor's contribution scores can be used to determine the prognosis of the patient. The 3-year and 5-year calibration charts (Figure 7(b)) showed that our nomogram performs well compared to an ideal model. The decision curve analysis shows that the clinical utility of our nomogram greatly overwhelmed the clinical features (Figure 7(c)). It showed that by using mitoRiskscore plus clinical features to predict prognosis, more patients could benefit from it.

### 3.9. In Vitro Verification of MitoRiskscore

**Figure 8:**
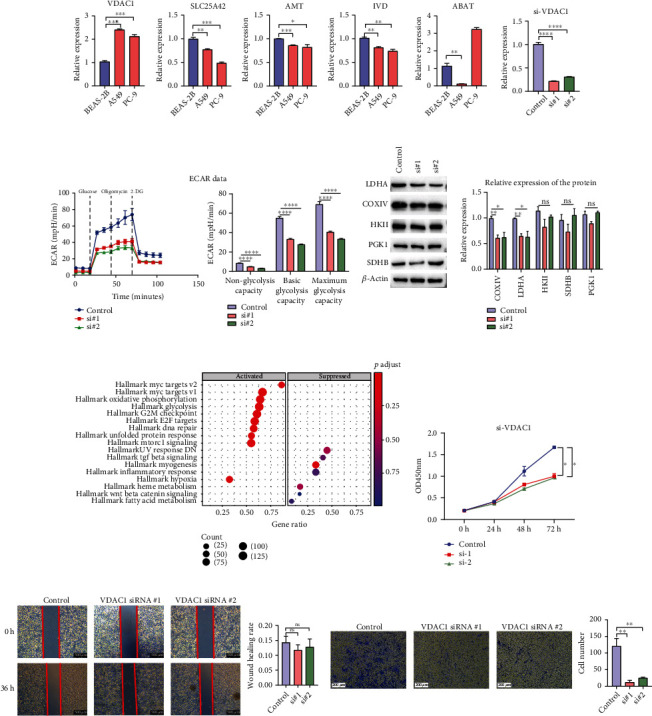
The regulation of the key genes in mitoRiskscore gene panel in the glycolysis metabolism, proliferation, and invasion of LUAD cells. (a) The expression of five genes in bronchial epithelial cell line (BEAS-2B) and lung adenocarcinoma cell lines (A549 and PC-9). (b) Knockdown efficiency of VDAC1 siRNA in A549 cell line. (c) Representative image and analysis of ECAR measurement in VDAC1 knockdown and control A549 cells. (d) Protein expression levels of LDHA, COXIV, SDHB, PGK1, and HKII in VDAC1 knockdown cells. (e) CCK-8 assays were used to evaluate A549 cell proliferation after VDAC1 knockdown. (f) GSEA analysis between patients with high and low expression of VDAC1. (g) The wound healing assay showed the migration ability of A549 cells after VADC1 knockdown. (h) Transwell experiments were performed to analyze the cell invasion ability after VDAC1 knockdown. ^∗^*p* < 0.05, ^∗∗^*p* < 0.01, ^∗∗∗^*p* < 0.001, ^∗∗∗∗^*p* < 0.0001.

To further verify the authenticity of mitoRiskscore, we conducted in vitro experiments on the five genes in mitoRiskscore. First, we used two lung adenocarcinoma cell lines (A549 and PC-9) and a bronchial epithelial cell line (BEAS-2B) to detect the expression of these five genes (Figure 8(a)). As expected, VDAC1 was highly expressed in the two LUAD cell lines and low in normal lung epithelial cells. The expression patterns of the other four genes were reversed. Given that the expression of VDAC1 accounted for the largest proportion in mitoRiskscore, we further conducted in vitro experiments to verify the function of VDAC1.

In order to explore the effect of VDAC1 on the metabolic ability of lung cancer cells, we measured the extracellular acidification rate (ECAR) to quantify the glycolysis ability in A549 cells knocking down VDAC1 in A549 cell line (Figure 8(b) and 8(c)). The results showed that when the expression of VDAC1 in lung cancer cells was downregulated, both the basic glycolysis level and the maximum glycolysis level decreased significantly. The glycolysis level of tumor cells is increased, suggesting that VDAC1 knockdown may inhibit tumor growth by inhibiting cell metabolism. Next, we tested the effects of knocking down VDAC1 on several key enzymes of glycolysis and oxidative phosphorylation in A549 cells (Figure 8(d)). Our results showed that the protein expression of cytochrome c oxidase IV (COXIV) and lactate dehydrogenase (LDHA) decreased after knocking down VDAC1. In contrast, there was no significant change in the protein expression of other enzymes such as succinate dehydrogenase complex subunits B (SDHB), hexokinase II (HKII), and creatine phosphoglycerol kinase 1 (PGK1).

Based on the expression of VDAC1, we divided the TCGA-LUAD samples into high expression (top 20%) and low expression groups (bottom 20%). GSEA was used to analyze the pathways activated in patients with high expression of VDAC1 compared to patients with low expression (Figure 8(e)). The results showed that in patients with high expression of VDAC1, the pathways that promote cell proliferation, such as MYC, MTOR, and energy metabolism, were significantly activated. CCK-8 assays indicated that the downregulation of VADC1 significantly reduced the proliferation ability of A549 cells (Figure 8(f)). Then, a transwell assay was performed to examine the effect of VDAC1 on the invasion ability of LUAD cells. The results showed that knockdown of VDAC1 significantly inhibited the invasion ability of A549 cells (Figure 8(h)). In contrast, we found through wound healing assay that the silencing of VDAC1 did not affect the migration of A549 cells (Figure 8(g)). Taken together, in vitro experiments confirmed that knocking down VDAC1 attenuated the ability of LUAD cells to glycolysis. At the same time, it can reduce the proliferation and invasion ability of LUAD cells.

## 4. Discussion

Abnormal metabolism is a very typical feature of cancer cells. Due to the central position of mitochondria in cell material metabolism, it is necessary for us to analyze the mitochondrial state of cancer. The most famous theory about the changes in mitochondria's energy metabolism in cancer was the Warburg effect put forward in the 20th century. That was, most cancer cells produced energy through glycolysis [20]. With the deepening of research, the Warburg effect was gradually overturned. It has been found that the primary way for cancer cells to produce energy was by aerobic oxidation, and the increase in glycolysis was to create more intermediate metabolites [7]. For example, it has been found that the colon cancer cell line SW620 had increased OXPHOS but glycolysis [8]. Ovarian cancer cells gave priority to OXPHOS to address energy requirements [21]. Besides, increased glycolysis does not always promote the development of cancer. In lung cancer cells, knockout of AIF increased glycolysis and reduced oxidative phosphorylation level but inhibited cancer cells' growth [22]. In addition to OXPHOS, cancer cells also showed an increased breakdown of fatty acids and amino acids. For example, *α*-ketoglutarate (an intermediate produced by the decomposition of glutamine) could supplement mitochondrial TCA intermediates [9]. The high metabolism of cancer cell mitochondria produced excessive ROS, promoting the death of normal cells, thereby promoting tumor progression [10]. In addition, the dynamics of cancer mitochondria were also changed. Mitochondria maintain their functions through autophagy, division, and fusion. The activity of mitochondria in cancer increased, and there were more mitochondria in a single tumor cell [23]. Therefore, our research focused on the changes in LUAD mitochondria.

We first analyzed the changes in mito-protein genes and metabolism in LUAD and normal lung tissues. Not surprisingly, both OXPHOS and glycolysis were very active in LUAD. But we also found that lipid metabolism was not active. Because the transcription of mito-protein genes was abundant in LUAD, we tried to find the relationship between them and patients' prognosis. Among the 1136 mito-protein genes, we found that 199 are significantly related to LUAD patients' prognosis, which was a large proportion. Through LASSO cox regression, we further screened five genes (VDAC1, SLC25A42, ABAT, IVD, and AMT) and constructed a prognostic panel named mitoRiskscore.

Among the genes of mitoRiskscore, VDAC1 is the most studied. VDAC1 is a transporter protein on the outer mitochondrial membrane (OMM) [24]. The functions of VDAC1 in metabolism and energy homeostasis are reflected by its facilitation of the transport of ions, nucleotides, and other metabolites across the OMM [25–27]. Specifically, VDAC1 is a transporter of cellular metabolites [25], cholesterol [28], lipids [29], and Ca2+ [30]. VDAC1 allows two-way traffic, mediating the entry of metabolites including pyruvate, malate, succinate, and NADH into mitochondria and the exit of newly formed molecules. The importance of VDAC1 in cell energy and metabolism homeostasis is reflected in the findings that downregulation of VDAC1 expression resulted in reduced metabolite exchange between mitochondria and the cytosol and inhibited cell growth, showing VDAC1 as essential for energy production and cell growth [31]. In our current research, we performed GSEA between the VDAC1 high expression group and the low expression group. The results showed that glycolysis, oxidative phosphorylation, MYC targets, E2F targets, and mTOR signaling pathways were all significantly activated in the high expression group. These activated pathways are related to tumor proliferation and metastasis. This result was consistent with our subsequent in vivo experiments. Because VDAC1 plays an important role in material transport in OMM, we believe that knocking down VDAC1 affects cell metabolism by restricting substance transport. At the same time, the expression of some metabolic enzymes decreased reflexively [32, 33]. We also found that knocking down VDAC1 could suppress the glycolysis metabolism, proliferation, and invasion of LUAD cells, which proved its momentous position in the progression of LUAD.

ABAT (4-aminobutyric acid aminotransferase) is responsible for the catabolism of *γ*-aminobutyric acid (GABA). GABA is an important inhibitory neurotransmitter in the central nervous system. ABAT deficiency phenotypes include psychomotor retardation, hypotension, hyperreflexia, lethargy, refractory epilepsy, and abnormal EEG. The ABAT gene is closely related to neurological diseases, but it is also associated with cancer. Our survival analysis showed that ABAT was a protective gene. The low expression of ABAT in basal cell-like breast cancer (BLBC) promoted the increase of GABA, thereby enabling the development and metastasis of cancer cells by activating the Ca2 ^+^ -NFAT1 axis [34]. In renal clear cell carcinoma, low-expressed ABAT promoted tumor development by boosting cancer cells' metabolism, which the transcription factor HNF4A may regulate [35]. ABAT was related to the prognosis of patients with liver cancer, pancreatic cancer, and breast cancer [14, 15, 36]. SLC25A42, AMT, and IVD genes have also been found in other prognostic models of lung cancer [37, 38]. However, no studies have found a relationship between AMT and cancers. Our research found that these three genes' high expression was related to LUAD patients' prognosis and was a protective factor. Further study of the specific mechanisms of these three genes inhibiting LUAD may provide new directions for LUAD treatment.

MitoRiskscore contains the above five genes, and its ability to predict the prognosis of LUAD patients is higher than that of clinical features (Figure 3). What is more commendable is that we have used a lot of external datasets to verify this conclusion (Figure 4). MitoRiskscore predicted LUAD patients' prognosis well in most external verifications, indicating that it is a reliable and effective prognostic evaluation tool. Next, we explored the specific biological significance of mitoRiskscore. High mitoRiskscore represents relatively active metabolism and high expression of mitochondrial localization protein genes (Figure 5). Specifically, the glycometabolism and OXPHOS pathways are significantly activated in LUAD with high mitoRiskscore, which indicates that cells have higher energy production and proliferation capabilities. However, we have also observed activation of the p53 pathway and nucleotide repair in high mitoRiskscore tumors, which may be feedback to the cell's high DNA replication and proliferation state. On the other hand, we found that lipid metabolism is relatively weak in high mitoRiskscore tumors. Fatty acid synthesis occurs in the cytoplasm. They are derived from acetyl-coenzyme A and are mainly provided by the tricarboxylic acid (TCA) cycle [39]. Based on the above results, we speculate that there is an upregulation of carbohydrate metabolism in lung cancer with highly activated mitochondria, which generates much energy through OXPHOS. The energy and acetyl-coenzyme A produced by these TCAs are not used to synthesize lipids. They are used by cells to maintain a high proliferation state and are associated with poor postoperative survival rates for patients. In short, mitoRiskscore can evaluate the energy metabolism patterns of the samples.

In this study, the R software package “pRRophetic” and mRNA expression profile were used to predict patients' six drug sensitivity and controls. This method required more clinical trial verification and only gave us a preliminary hint (Figure 6). The results showed no difference in the sensitivity of each group to vinorelbine, and even the control group was more sensitive to cisplatin. Different samples in the same group had different sensitivity to etoposide and paclitaxel. The high mitoRiskscore group's sensitivity to docetaxel and gemcitabine was higher than that of the low mitoRiskscore group, and both groups were higher than the control group. Furthermore, the sensitivity of patients to docetaxel and gemcitabine was significantly negatively correlated with the mitoRiskscore. It showed that the use of docetaxel and gemcitabine to treat LUAD with active mitochondrial function might achieve better results.

We constructed a nomogram containing mitoRiskscore and clinical features, making the results of this study easier to apply to the clinic (Figure 7). Nomogram is a popular tool for predicting the prognosis of cancer. It uses statistical methods to combine patients' parameters to predict their prognosis. Due to a combination of factors, the accuracy of nomogram is more accurate than that of a single stage of cancer [19, 40]. The calibration chart analysis and the decision curve analysis showed that the nomogram had better prediction accuracy and can benefit more patients.

One of the innovations of our research is that we developed a LUAD prognosis predictive model based on mitochondrial localized genes for the first time. Since mitochondria are involved in most of the metabolic pathways, our model is more representative than the general metabolic model. In addition, our model screened several genes that have not been studied in the field of lung cancer but are closely related to the prognosis of LUAD patients, such as SLC25A42, AMT, and IVD, which provides a basis for future research on the mechanism of lung cancer.

There were still some limitations in this study. Firstly, the transcriptome data used for panel construction comes from sequencing. Although we had verified the function of mitoRiskscore using chip data, PCR data has not been used yet. Secondly, since this study used gene expression data as categorical variables to be input into Cox regression, it is necessary to further determine the optimal cut-off value. Thirdly, because the study was retrospective, the patient population is heterogeneous, and the results may be biased. The mitoRiskscore and nomogram obtained in this study require more clinical studies to verify their effectiveness.

## 5. Conclusions

In conclusion, the mitoRiskscore gene panel is an effective tool for the survival prediction of LUAD patients. It can also assess the metabolic status of tumors and assist with clinical chemotherapy. However, further clinical trials are needed to verify our findings.

## Figures and Tables

**Table 1:** Basic information of datasets used in this study.

Datasets	Platform	Country	No. of patients	No. of controls	Cancer type	Prognostic information
GSE7670	GPL96	China	31	27	LUAD	—
GSE18842	GPL570	Spain	46	45	NSCLC	—
GSE19188	GPL570	Netherland	45	65	LUAD	—
GSE31210	GPL570	Japan	226	20	LUAD	Yes
GSE3141	GPL570	USA	58	—	LUAD	Yes
GSE8894	GPL570	South Korea	63	—	LUAD	Yes
GSE50081	GPL570	Canada	127	—	LUAD	Yes
GSE68465	GPL96	USA	442	—	LUAD	Yes
GSE72094	GPL15048	USA	398	—	LUAD	Yes
TCGA-LUAD	IlluminaHiSeq	USA	513	59	LUAD	Yes

LUAD: lung adenocarcinoma; NSCLC: non-small-cell lung cancer; TCGA: The Cancer Genome Atlas.

## Data Availability

The self-composed running scripts, together with the processed results of current study, were merged into a repository that is available at https://github.com/XR-Zhang-group/mitoRiskscore. The data used to support the findings of this study are also available from the corresponding author upon request.

## Supplementary Materials

Supplementary Materials Supplementary Figure S1: forest plot of the univariate Cox regression analysis with the expression of genes encoding mitochondrial localization protein.
