# Supplementary figures and images for: A Robust Panel Based on Mitochondrial Localized Proteins for Prognostic Prediction of Lung Adenocarcinoma
Source: Oxid Med Cell Longev. 2021 Sep 9;2021:7569168. doi: 10.1155/2021/7569168 (PMC8445726; doi:10.1155/2021/7569168)

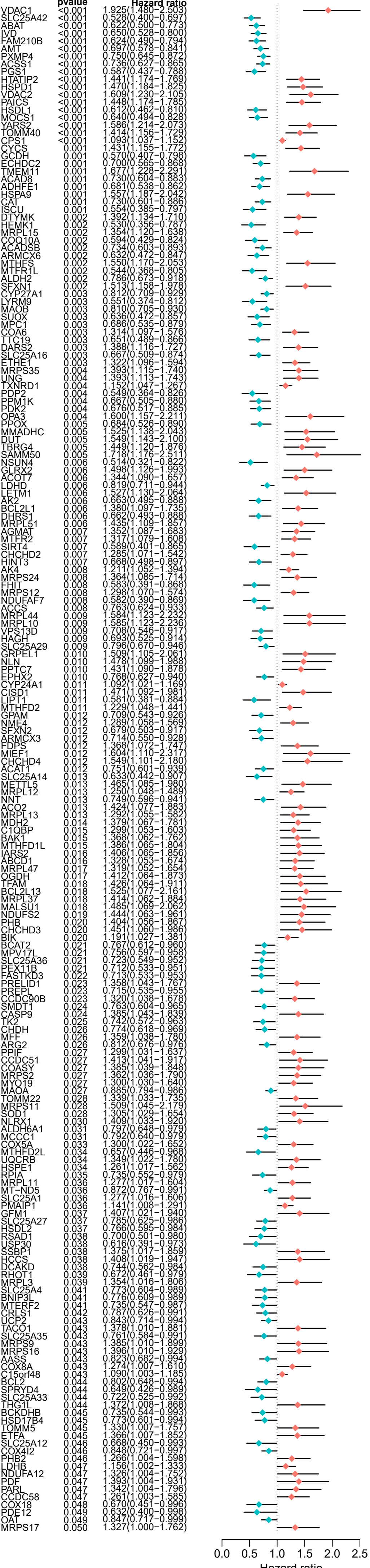

0.0 0.5 1.0 1.5 2.0 2.5  
Hazard ratio

Supplement: Supplementary Materials — Supplementary Figure S1: forest plot of the univariate Cox regression analysis with the expression of genes encoding mitochondrial localization protein. [file 7569168.f1.pdf]
